# Exploring food security/insecurity determinants within Venezuela’s complex humanitarian emergency

**DOI:** 10.1016/j.dialog.2022.100084

**Published:** 2022-11-21

**Authors:** Marianella Herrera-Cuenca, Maritza Landaeta-Jiménez, Pablo Hernandez, Yaritza Sifontes, Guillermo Ramírez, Maura Vásquez, Thais Maingon

**Affiliations:** a Center for Development Studies (CENDES), Central University of Venezuela, Neverí Av., Fundavac Building, Colinas de Bello Monte. ZIP: 1080, Caracas, Venezuela; b José María Bengoa Foundation for Food and Nutrition, Centro Seguros La Paz Building, 4th floor, Office E-41C, La California sector, Francisco de Miranda Av., ZIP: 1071, Caracas, Venezuela; c Venezuelan Health Observatory, Neverí Av., Fundavac Building, Colinas de Bello Monte. ZIP: 1080, Caracas, Venezuela; d Nutrition and Dietetics School, Central University of Venezuela, Address: El Rectorado Av. Res. 2-B, 3th floor, University City of Caracas, Los Chaguaramos. ZIP: 1041-A, Caracas, Venezuela; e Postgraduate Area in Statistics, Universidad Central de Venezuela, Address: El Rectorado Av. Res. 3-A, 3th floor, University City of Caracas, Los Chaguaramos. ZIP: 1041-A, Caracas, Venezuela; f Metropolitan University. Address: Distribuidor Universidad. Boyacá Av. with Petare-Guarenas highway. Terrazas del Ávila Urb., Caracas, -Miranda State. ZIP: 1073. Caracas, Venezuela

**Keywords:** Food security, food security determinants, Venezuela, Complex Humanitarian Emergency, CARI Console, Food Security Index, CHAID, COVID-19

## Abstract

**Introduction:**

The complex humanitarian emergency that Venezuela has been going through for several years has deteriorated the quality of life of its citizens, deepened food insecurity in households and has promoted migratory movements of almost six million people to neighboring countries.

**Objective:**

To analyze food security in Venezuelan households to identify the determinant factors that might contribute to the design of evidence-based public policies.

**Materials and methods:**

A non-probabilistic survey of national scope was used in 2,041 urban and non-urban households. A descriptive statistical test was performed to analyze demographic variables and the three component indicators of the food security index (FSI): food consumption, economic vulnerability and coping strategies. The FSI was built according to the World Food Program (WFP) methodology, and a segmentation analysis was applied using the Chi-squared Automatic Interaction Detection (CHAID) algorithm to specify the influence of some variables as the best predictor at each level.

**Results:**

Only 9% of the households presented food security, 69% classified as marginally secure, and 22% presented moderate or severe food insecurity. The food consumption score (FCS) was the variable that best discriminated the level of food security, followed by coping strategies and the percentage of spending on food. **Conclusion:** Most of the households studied sacrifice their livelihoods to feed themselves and cover the minimum of their nutritional requirements. This needs attention to stop and reverse the deterioration within a framework of respect for the human rights to health and food.

## Highlights

• This research shows the precarious conditions of food security in the studied population.
• 22% of Venezuelan households reported to be in moderate and severe food insecurity.
• Only 9% of the households presented food security, and 69% were classified as marginally secure.
• The food consumption score was the variable that best discriminated the food security index.
• Households sacrifice their livelihoods to feed themselves and cover their requirements.

## Graphical Abstract

**Figure:**
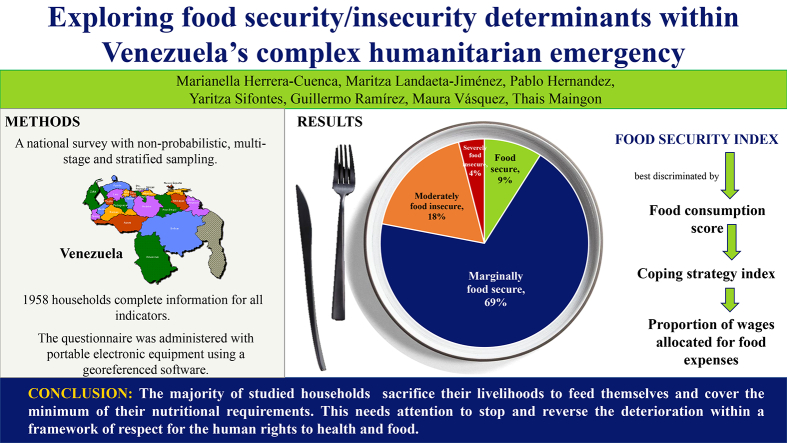

## 1 Introduction

Food and nutritional security (FNS) refer to the sufficient supply in quantity and quality of food that is safe, nutritious and consistent with the preferences or socio-cultural acceptability of all people. It includes stability in prices, delivery and access, means of production, the capacities of households to utilize food at all times, physically, socially and economically, and how they use it to satisfy their nutritional needs in such a way as to guarantee their growth, healthy development, and active and productive life [[1], [2], [3]]. Thus, its multidimensional character is given by the relationship between food and the economy of the countries, in addition to agro-production, and the four pillars that define it: availability and accessibility, bio-utilization and stability [4].

Measuring food security should include identifying the affected people, assessing the severity and nature of the problem, analyzing the trends, and providing a foundation for evaluating the impact on the population, all of which constitute the basis for decision-making aimed at improving the situation if necessary [5]. The indicators selected for this purpose must reflect the four components of the FNS mentioned above [6]. Among the methods for assessing food security are at least five: a) the United Nations Food and Agriculture Organization (FAO) estimate of calories per capita available at the national level; b) household income and expenditure surveys; c) food intake of individuals; d) anthropometry and e) perception-based food insecurity (FI) measurement scales [1,7,8].

In the search to improve the monitoring of the food situation and specifically undernourishment, indices have emerged, such as the Global Index of Family Food Security (GIFFS) or the FAO prevalence and magnitude of undernourishment index [6]. Various scales have also been used; one of the best known is the Food Insecurity Experience Scale (FIES), which measures the access of individuals or households to food. It is based on two other scales: the US Household Food Security Survey Module (HFSSM), carried out since 1995 by the United States Department of Agriculture, and the Latin American and Caribbean Food Security Scale (ELCSA). The FIES asks about eating experiences and behaviors related to resource limitations that prevent access to food, considering three levels: uncertainty/concern, changes in food quality, and changes in food quantity [9].

The World Food Program (WFP) proposes an evaluation of food security based on constructing an index that integrates food consumption, survival strategies associated with livelihoods and economic vulnerability. In Latin America, the unequal region in the world before the pandemic in 2019 [10], some 47.7 million people experienced hunger, and the situation worsened in the last 5 years due to the increase of 13.2 million undernourished people [11], of these, the increase of undernourishment in the Bolivarian Republic of Venezuela is significant. The State of Food Security and Nutrition in the World 2019, indicates that the rate of undernourishment in Venezuela almost quadrupled, going from 6.4% in the period 2012-2014 to 21.2% in 2018, therefore the number of hungry people in the country rose from 2.3 to 6.8 million. The increase in undernourishment in Venezuela in 1.3 million more people in the 2014-2016 period compared to the previous three years, explains an important issue of the incremented numbers experienced in this indicator in South America lately [[11], [12], [13]].

Venezuela adds to the previous problem, the adaptation of the most vulnerable population to lower availability of food and access to it through coping strategies such as reducing the amount of food eaten, eliminating meals and substituting preferred food for others. The gaps between the different social levels are widening while the right to food is systematically violated. In general, international agencies have documented the deterioration in the Venezuelan diet and its consequences on the nutrition and health of the population [11,12].

The COVID-19 pandemic took the country in a severe crisis of public services; among them, one of the most important is the lack of water within households, which also coexists with food insecurity. This key element, increases nutritional vulnerability because it can cause malnutrition, psycho-emotional stress, and increased risk of infectious and chronic diseases, which occurs through multiple pathways, including poor nutrition and diseases caused by inadequate environmental sanitation [14]. These are factors that aggravate food insecurity in Venezuelan households and maintain the high morbidity that has been deteriorating the health of children and mothers, especially during the first 1,000 days of life, increasing acute malnutrition and mortality among infants [15,16].

Venezuela ranked seventh in 2021 on the map of the 20 countries with a "high risk" of facing acute food insecurity, preceded by the Democratic Republic of the Congo, Afghanistan, Yemen, Nigeria, Ethiopia and Syria, South Sudan, Haiti and Guatemala that complete the first 10 places, in that exact order [17], this is consistent with the raising in food insecure people reported in the country [18]. In addition, different national organizations coincide in showing the increase in cases of acute undernutrition as a result of the situation in which the country finds itself due to the overlapping of two crises: the pre-pandemic and the ongoing pandemic [19]. All of these occurring in the realm of an accelerated process of dollarization of goods but not of the wages and formal income [20].

In accordance with the previous context, the objective of this work is to analyze the food security of Venezuelan households to identify determinant factors that allow the design of evidence-based public policies.

## 2 Materials and methods

### 2.1 Sample and study design

A non-probabilistic, multi-stage and stratified sampling by federal entities with allocation proportional to the geographical location was carried out. The estimated size of the sample was 2,000 households, equally distributed according to the type of area (1,000 urban and 1,000 non-urban). The allocation of the sample in the strata is proportional to the population projected by the National Institute of Statistics (INE) of the entities for 2020 [21]. The fieldwork was carried out nationwide between December 2020 and February 2021. The final sample was 2,041 households (1,023 urban and 1,018 non-urban), of these subjects, only 1,958 presented complete information for all indicators.

### 2.2 Collection of information

The study was conducted in accordance with the Declaration of Helsinki, and its protocol was approved by the ethics committee of the Central University of Venezuela (04-20-2021). It should be noted that due to COVID-19 restrictions, a previous e-mail authorization was received in order to start the data collection. However, the official approval letter was received physically at a later date; because of delays in ordinary procedures.

With prior digital consent to participate in the study, in each surveyed household, the head of the family was selected to respond to the interview, which was conducted by a well-trained interviewer. The questionnaire was based on the WFP data collection instrument for socio-economic conditions and food security in emergencies adapted for Venezuelan foods [2]. The CARI console (Consolidated Approach Reporting Indicator) was developed, including three main domains: food consumption, economic vulnerability, and coping strategies [22]. By combining the respective domains, a summary indicator called "Food Security Index" (FSI) was generated, representing the population's general state of food security. The questionnaire was digitized in georeferenced software (Survey123® by Esri®) and was administered with portable electronic equipment through an app developed especially for this study. This made it possible to obtain information in real time, locate the surveyed household through geographically location referenced methods (georeferenced, GPS), and reduce collection and transcription errors

### 2.3 Quality control

The interviewers and coordinators were trained in using the georeferenced mobile application and the procedure for obtaining the information. Strict quality control was performed to minimize errors in the data collected.

### 2.4 Data and indicator collected

#### 2.4.1 Sociodemographic

In this category, the included indicators were: type of parish (urban or non-urban), sex and age of the head of the family, marital status, number of household members and percentage of household members by sex.

#### 2.4.2 Food consumption

The Food Consumption Score (FCS), a proxy indicator representing the current diet's diversity, was used. To obtain this score, the number of days of the last week (7 days) in which the households consumed different food groups was inquired. Food was then categorized, considering the score obtained by assessing the frequency of consumption, the nutritional importance of the group, as well as the total variety of groups eaten by the household. The cut-off points of the WFP score were applied [2]: poor: 0 to 28 points; limited: 28.5 to 42 points and acceptable: >42 points.

#### 2.4.3 Economic vulnerability

The proportion of wages allocated for food expenses (PWFE) was used for this dimension. This indicator is based on the premise that the more critical food is within the general household budget (relative to other items/services consumed), the more economically vulnerable the household is. This indicator is constructed by dividing total food spending by total household spending. The cut-off points established by the WFP for the four categories of this indicator are: less than 50%, between 50 and 65%, between 65 and 75% and more than 75% [2].

#### 2.4.4 Livelihood coping strategies

The coping strategy index -CSI- used is derived from a series of questions about the household's experiences with livelihood stress and asset depletion during the 30 days prior to the survey. The answers are used to understand the stress and insecurity that households face and describe their capacity for productivity in the future. These strategies are classified into three groups, organized in ascending order according to the intensity of their effect on livelihoods and asset depletion: stress, crisis and emergency.

Stress strategies indicate a diminished capacity to face crises in the future due to the current reduction of resources or increase in debts. Crisis strategies, such as selling productive assets, directly reduce future productivity, including human capital formation. In addition to affecting future productivity, emergency strategies are irreversible and indicate the depletion of household resources. Table S1, in the supplementary material, shows the coping strategies selected in each category.

Households that do not carry out any of the aforementioned strategies are households with food security. Those who apply stress strategies are households with marginal food security, those who use crisis strategies are in moderate food insecurity, and those who use emergency strategies are severely affected by food insecurity. It is important to clarify that, according to this methodology, each household is classified according to the most severe coping strategy reported [2].

#### 2.4.5 Food security index (FSI)

The Food Security Index (FSI) is obtained by combining the three indicators evaluated: food consumption score, economic vulnerability, expressed by the proportion of spending on food, and livelihood coping strategies, which are round to the nearest integer. The index offers four categories according to the score: Food Security (FS), Marginal Food Security (MFS), Moderate Food Insecurity (MFI) and Severe Food Insecurity (SFI) [22].

#### 2.4.6 Data analysis

For the descriptive analysis, absolute and relative frequency distributions were used, as well as the mean and standard deviation, for the demographic variables. A chi-squared test was used to evaluate statistical independence between the food security index (FSI) and its the three component indicators: food consumption, food expenditure, and coping strategies, so together them. The the influence of the component indicators on the FSI performance was evaluated using cross tables and chi-square tests. Next, a segmentation analysis was performed using the CHAID (Chi-Square Automatic Interaction Detection) algorithm. This technique divides the population into two or more different groups according to the categories of the dependent variable considered to be the best predictor at each segmentation level. In all cases, the tests were performed with a statistical significance level of 0.05. Microsoft Excel® 2016 software and the IBM SPSS® version 23 statistical package was used for data loading and analysis.

## 3 Results

From the overall recruited sample of 2,041 (1,023 urban and 1,018 non-urban), 1,958 were obtained with all the required information (FCS, CSI, and PWFE). Thus, total *n* value for building the FSI was *n*=1958.

Table 1 shows the sociodemographic characteristics of the sample. The majority of households head were females (56.9%) compared to males (43.1%). The average age of household heads was 48.7 ± 21.5. 50.8% % of these heads of household are married or live with a partner.

**Table 1:** Socio-Demographics characteristics.

Variable	Categories	n	%
Gender of the household head	Female	1162	56.9
	Male	879	43.1
Age of the household head		48.7 ± 21.5	
Civil status of the household head	Married or as a couple	1038	50.8
	Divorced or widowed	477	23.4
	Single	526	25.8
Household member size	<3	738	36.1
	3-6	1213	59.4
	>6	90	4.5

The Food Security Index (FSI) for Venezuela 2020 presented in the CARI Console (Table 2) indicates that very few of the households participating in the study can be considered in FS status (9%), the vast majority are in MFS (69%), a smaller number of households present MFI (18%), and very few are in SFI (4%). FCS in most households corresponds to acceptable consumption (85%), a small group of households have limited consumption (11%), and only a few households have a poor level of consumption (4%). Regarding food expenses, a little less than a third of the households (29%) have a PWFE < 50%. In the rest of the households, an important group stands out (40%), with food expenses exceeding 75% of the total expense. The coping strategy index as a percentage of coping strategies (CSI) applied shows that there are very few households that do not use coping strategies or apply only stress strategies (11%), while an extensive majority use more severe strategies, such as crisis (58 %) or emergency (21%). Table 2 reports all the above mentioned results.

**Table 2:** Consolidated approach for reporting indicators of food security (CARI) reporting console. (Percentage by rows).

Domain		Indicator	Food secure (FS) %	Marginally food secure (MFS) %	Moderately food insecure (MFI) %	Severely food insecure (SFI) %
Current status	Food Consumption	Food consumption score (FCS) n = 2028	85	-	11	4
Coping Capacity	Economic Vulnerability	Proportion of wages allocated for food expenses (FWFE) n = 1964	29	17	14	40
	Livelihood coping strategies	Coping strategy index (CSI) n = 2041	10	11	58	21
Food security index n = 1958			9	69	18	4

### 3.1 Association between food security and its components

A significant association (by Chi-square test) between the the indicators that define food security index (FSI) food consumption, (FSC), Proportion of wages allocated for food expenses (PWFE) and Coping Strategy Index (CSI) was found (Table 3). Regarding the FCS, almost all the households in FS and MFS have an acceptable FCS; in households with MFI, the acceptable FCS (40%) decreases significantly, finding instead a limited FCS (53%), and only a small group of households with poor FCS (7%). The chi-square association between the total FSI for all levels and FSC was highly significant (p < 0.001). In households with SFI, consumption is reduced; all households have a limited FCS (46%) or poor (54%). Regarding the association between the total FSI and the PWFE (p < 0.001), households with FS do not commit the total expenditure for the purchase of food (PWFE < 50%: 82.5%). In households with MFS, total spending is more affected by food expenses (PWFE > 50%: 73%); and in households with MFI and SFI, total spending is highly impacted by food spending, with levels of PWFE > 65% (76%) and PWFE > 75% (91%) being found in these two groups, respectively. In what corresponds to the association between the total FSI and the CSI (p < 0.001), most households with FS do not apply coping strategies (66%). Households with MFI mainly use crisis strategies (72%); households with MFI apply higher impact strategies corresponding to crisis (42%) or emergency (49%) levels; and the small group of households in SFI mostly use more inclement strategies, crisis (38%) or emergency (56%).

**Table 3:** Food security association with its components (Percentage by columns).

Variable	Categories	Food security index				Total
		FS	MFS	MFI	SFI	
Food consumption score	Acceptable	100.0	99.5	40.1	0.0	85.0
	Borderline	0.0	0.5	52.8	46.4	11.5
	Poor	0.0	0.0	7.1	53.6	3.5
Proportion of wages allocated for food expenses	<50%	82.5	26.9	14.7	0.0	28.9
	50–65%	17.5	20.5	9.4	1.2	17.5
	65–75%	0.0	16.5	11.8	8.3	13.8
	>75%	0.0	36.1	64.0	90.5	39.9
Coping strategy index	None	65.6	5.3	4.7	0.0	10.6
	Stress	34.4	10.1	4.1	6.0	11.1
	Crisis	0.0	71.7	42.2	38.1	58.5
	Emergency	0.0	12.9	49.0	56.0	19.8

FS = Food secure (n=183); MFS = Marginally food secure (n=1352); MFI = Moderately food insecure (n=339); SFI = Severely food insecure (n=84).

### 3.2 FSI segmentation according to indicators

**Fig. 1:**
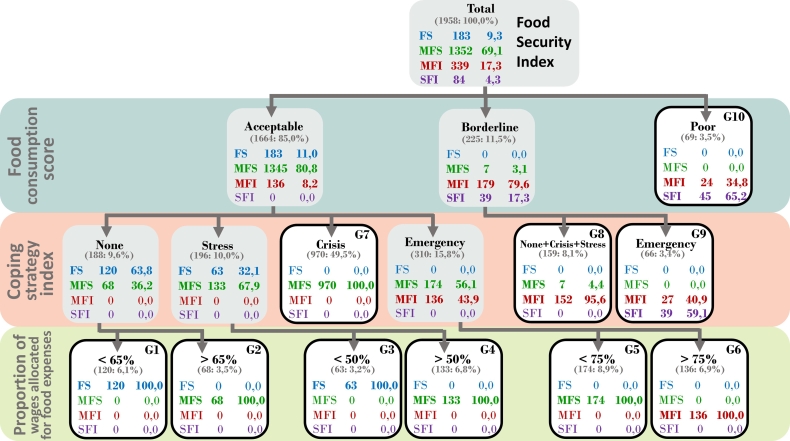
Household segmentation tree by FSI, according to FCS, CSI and PWFE. FSI=Food Security Index; FCS=Food Consumption Score; PWFE=Proportion of Wage allocated on Food Expenses; CSI= Coping Strategy Index; FS = Food secure; MFS = Marginally food secure; MFI = Moderately food insecure; SFI = Severely food insecure.

The CHAID segmentation procedure applied to 1958 surveys which included all the indicators of the FSI, leaving 83 lost due to missing values, evaluate the impact produced jointly by the FCS, and PWFE and CSI indicators on the FSI, manages to identify the FCS at the first level of segmentation as the indicator with the most significant capacity to discriminate between households. At the second level, the indicator with the most significant potential for segmentation is the CSI, both in households with acceptable FCS and those with limited FCS. In households with poor FCS, segmentation does not occur, and finally, in the third level, the PWFE produces a segmentation, giving rise to terminal groups described according to the FSI category (Fig. 1).

Households in FS condition (n= 183), identified by groups G1 and G3, have an acceptable FCS in common; CSI and PWFE determine their differences. The group of households G1 (120 households) does not apply coping strategies; in all households, the total expenditure for the purchase of food is impacted to some degree (PWFE: < 65%). In the G3 group, all its members (63 households) use stress strategies without affecting the PWFE: < 50%.

Households in MFS condition (n = 1352), made up of four groups G2, G4, G7 and G5, which share the particularity of an acceptable FCS, present as a characteristic a marked interaction between CSI and PWFE, in these households. In general, maintain a level of consumption of acceptable food, involved establishing an inverse relationship between the severity of the coping strategies used and the amount corresponding to spending on food, as part of total spending (Table 4): G2, 68 households, do not apply coping strategies, but almost all of them require high levels of PWFE 65-75% (24%) and PWFE>75% (71%); G4, 133 households, which use stress strategies, with a high group of households whose total spending is affected by food spending: PWFE 50-65% (35%), PWFE 65-75% (16%) and PWFE >75% (47%); G7, 970 households, which use more rigorous strategies corresponding to the crisis level, with high contingents of households that commit an important part of the total expenditure on food (PWFE > 50%: 71%); G5, 174 households, employing emergency coping strategies, with approximately half of the households not affecting their PWFE <75%.

• Marginally food secure (MFS) households: n=1352

**Table 4:** Distribution of the Proportion of Wage allocated for Food Expenses (PWFE) in marginally food secure (MFS) households by coping strategies

Coping Strategy Index		Proportion of wages allocated for food expenses				Total
		<50%	50–65%	65–75%	>75%	
None	n	4	0	17	51	72
	%	5.6	0.0	23.6	70.8	5.3
Stress strategies	n	3	47	22	64	136
	%	2.2	34.6	16.2	47.1	10.1
Crisis strategies	n	276	176	145	373	970
	%	28.5	18.1	14.9	38.5	71.7
Emergency strategies	n	81	54	39	0	174
	%	46.6	31.0	22.4	0.0	12.9
Total	n	364	277	223	488	1352
	%	26.9	20.5	16.5	36.1	100

Households in MFI condition (n= 339) are composed of two groups: G6, 136 households, with acceptable FCS, which apply emergency-level coping strategies, with a high PWFE >75%; G8, 159 households, with a limited FCS, that in general do not apply coping strategies, or apply those that are less harsh in terms of stress or crisis level, are also households that do not differ in terms of PWFE levels.

Households in SFI condition (n= 84) comprise the groups: G9, 66 households with a limited FCS, apply emergency coping strategies without being able to differentiate themselves in terms of PWFE; G10, with a poor FCS, cannot differentiate themselves in terms of the coping strategies they use, nor concerning the PWFE.

## 4 Discussion

In this study, only 9% of households presented food security, while 69% presented marginal food security, with great vulnerability to falling into food insecurity, and 22% of households found themselves in moderate and severe food insecurity. The food consumption score (FCS) was the variable that best discriminated the food security index, followed by coping strategies. In 40% of households, spending on food exceeded 75% of total monthly spending, and 79% of households used crisis or emergency coping strategies to feed themselves. Households with SFI presented high vulnerability because they applied extreme coping strategies of crisis (38%) and emergency (56%) to face food insecurity. In addition, another factor that increases food vulnerability is that 57% of heads of households are women.

A study in rural areas of Zimbabwe [23], reports a CARI console that shows 41% of households in food security, 34% of households were marginally food secure, 21% moderate food insecure and 4% severe food insecure, whereas this study (urban and non-urban population) shows 9% of food security, 69% of households live in marginally food secure conditions, 18% are moderately food insecure and 4% experience severe food insecurity. The differences between both countries are interesting as rural areas might have more elements on family agriculture, self-production and more availability on some foods, depending on the crops.

The results pointed to the difficult situation of food insecurity in the households of this sample, which concur with other investigations carried out in the country since the complex humanitarian crisis was recognized in the last five years [24,25]. The situation can be worsened due to hyperinflation, failures in public services, economic restrictions and the dismantling of public institutions. In addition, the impact of restrictive measures due to COVID-19 and fuel shortages, have affected agricultural and productive activities [17,26,27]. Also, in these circumstances, the Venezuelan Food System is unable to guarantee the availability of food in sufficient quantity and quality, culturally acceptable and with sustainable access so that people have adequate food for their nutritional requirements [28].

If the FCS, the variable that best discriminates the FSI, is low, it demonstrates the ability of these households to face limitations in access to food, activating compensatory coping strategies to provide food to the detriment of other basic needs such as health and education [24,25]. In 9% of households with food security, the FCS is adequate in 100% of households, and the PWFE in 85.2% of households is less than 50%, and 34.4% of households use stress strategies to compensate for eating. On the contrary, in 50% of households with MFI, consumption is highly compromised, and in SFI households, consumption of foods is extremely precarious, thus affecting the FCS. At both levels of food insecurity, crisis and emergency coping strategies prevail. According to Béné *et al*. [29], this behavior is explained because households are not passive agents but rather respond to the risks and adverse events to reduce their vulnerability. For his part, Maxwell *et al*. [30] points out that as food insecurity worsens, coping strategies become irreversible.

The households that use irreversible coping strategies, such as selling their house, car or productive assets, in the middle and long term are exposed to further deterioration of their already poor condition of food security, which increases hunger and social conflict [31]. In countries with protracted crises, survival strategies constitute the main means of coping with emergency times. However, when these strategies fail, food insecurity accelerates, hunger and social conflict increase, and the environment becomes adverse; the overall situation negatively affects the welfare of the population [7,32]. Indeed, the sudden increases in food prices in 2007-2008 and 2010-2011 in the world coincided with large-scale demonstrations and incidents of violence that evidenced the link between food insecurity and social conflicts [14].

In the households studied, the CSI and PWFE indicators interact markedly. It is observed that as food insecurity deepens, the food consumption is limited and strategies for optimizing feeding appear to preserve the purchase of food, but when the most extreme of those strategies such as those of crisis or emergency must inevitably be applied, the expenses on food increase and might deplete the income of the household [33].

Inflation has a deleterious effect on food security and has had a significant impact on the rise in food prices, which, according to the Central Bank of Venezuela at the end of 2021, stands at 1,575.26% [34]. In 40% of the households in the study, it was observed that spending on food exceeded 75% of the total monthly spending, meaning that in this group, everything is reduced to providing food. In addition, the percentage of spending on food is found in the third branching level of the segmentation tree, which divides the group and establishes the majority of the final nodes. It is considered that, in normal situations, spending on food is usually in the order of 20 to 30% of total monthly spending [35].

Ensuring a healthy diet in Venezuela at this time implies a highly complex challenge. The reduction of purchasing power, the internal (domestic) increase in imported food prices and the interruption in food distribution chains would further affect food spending and consumption, deepening food insecurity. The country's risk in the middle of the consequences of the pandemic would rise with an increase on informal employment, thus decreasing family incomes and leaving more people without social security benefits. Therefore, an additional increase in inadequate food consumption and poor quality would not be surprising [36].

Although there are natural resources and trained personnel in Venezuela, the urgent adoption of humanitarian interventions is required to establish a base to stop the deterioration while reactivating national production and other public policies that provide sustenance and opportunities to households while building strong food and nutrition institutions over the long term. The results of this study constitute a contribution to nutritional epidemiology in Venezuela, where each year, it is more challenging to access national figures or statistics necessary for the formulation of public policies. This research provides information on the serious situation faced by Venezuelan households due to the diversity of factors that harm food security. At the same time, they contribute to the planning of future actions aimed at transforming the situation described.

## 5 Limitations and strengths

Among the study’s limitations is the fact that in spite of being a study of national scope, it was limited to the states’ capitol (urban and non-urban areas near the capitol city) without la delimitation for socioeconomic statuses. The study’s frame period included December, which is the time of Christmas holidays, when Venezuelan families traditionally are concerned on how to provide more income to cover the traditional foods. However the data shown on this same study report the difficulties for accessing to foods, as households in moderate food insecurity and severe food insecurity expend more than 65% of their income in foods, only to achieve a monotonous and low quality diet. Yet, this study has important strengths such as the national outreach in a critical period within the Venezuelan crisis, that allowed to understand more on what was going on at national level, and the applied WFP- CARI methodology which is a complex method that integrates food consumption with means of life and copying strategies, that allows to better understand the difficulties of the food insecurity experience.

However, further studies must be carried out to identify possible changes in people's health and nutrition and analyze the impact that COVID 19 would have on the already compromised food security of the Venezuelan population.

The results of this study should be interpreted with caution because although the sample of households has a national scope, it is limited to the capitals of the states. Therefore, inferences should not be made beyond the sample analyzed.

## 6 Conclusions

This research shows the precarious conditions of food security in the studied population and the great effort that Venezuelan households are doing to provide food. The food consumption score was the factor that better assessed the food security total score within households, followed by the survival strategies and the percentage of income spent on food. This demonstrates the convergence of multiple factors in shaping food security, all of which must be taken into account within a framework of respect to the human rights to health and food.

## Funding

The funds provided by the Venezuelan Health Observatory-Bengoa (VHO-B) were only to pay for logistics of the project and the VHO-B is a non-profit organization that supports research and humanitarian assistance.

## Declaration of Competing Interest

The authors declare that they have no known competing financial interests or personal relationships that could have appeared to influence the work reported in this paper.

## Appendix A Supplementary data

Supplementary material Image 1
