# Supplementary material for: Exploring food security/insecurity determinants within Venezuela’s complex humanitarian emergency
Source: Dialogues Health. 2022 Nov 21;1:100084. doi: 10.1016/j.dialog.2022.100084 (PMC10954010; doi:10.1016/j.dialog.2022.100084)
Supplement: Supplementary file 1 — Supplementary material [file mmc1.docx]

**SUPPLEMENTARY MATERIAL**

**Exploring food security/insecurity determinants within Venezuela’s complex humanitarian emergency**

**Supplementary Table S1.** Livelihood coping strategies evaluated.

| **Categories** | **Livelihood coping strategy** |
| --- | --- |
| Stress  Strategies | Using credit for buying foods |
|  | Borrowing money for buying foods |
|  | Buying foods on a daily basis |
|  | Expend savings for buying foods |
|  | Extension of breastfeeding period to children. |
|  |  |
| Crisis  Strategies | Food barter |
|  | Sending household members to eat at community kitchens |
|  | Diminishing education and health expenses |
|  | Selling household goods |
|  | Selling productive actives |
|  | Selling household cars |
|  | Looking informal jobs |
|  | Working with payments in foods |
|  |  |
| Emergency  Strategies | Selling house or lands |
|  | Remove children from school |
|  | Taking risky jobs |
|  | Begging for money on the streets |
|  | Looking for leftovers within the garbage |
